# Dietary Supplementation with Encapsulated or Non-Encapsulated Sodium Butyrate Enhances Growth, Antioxidant Defense, Immunity, and Gut Health in Largemouth Bass (*Micropterus salmoides*)

**DOI:** 10.3390/microorganisms13071594

**Published:** 2025-07-06

**Authors:** Minghui He, Zhiwei Zou, Wanjia Zhu, Haipeng Li, Ting Liang, Liwei Liu, Jianmei Su

**Affiliations:** 1 Hubei Key Laboratory of Regional Development and Environmental Response, Faculty of Resources and Environmental Science, Hubei University, Wuhan 430062, China; 18380305308@163.com (M.H.); 202421108012111@stu.hubu.edu.cn (Z.Z.); 202321108012137@stu.hubu.edu.cn (W.Z.); 202421108012161@stu.hubu.edu.cn (H.L.); 202231108031008@stu.hubu.edu.cn (T.L.); 2 College of Fisheries, Chinese Perch Research Center, Engineering Research Center of Green Development for Conventional Aquatic Biological Industry in the Yangtze River Economic Belt, Ministry of Education, Huazhong Agricultural University, Wuhan 430070, China; liuliwei@mail.hzau.edu.cn

**Keywords:** *Micropterus salmoides*, encapsulation, sodium butyrate, growth performance, antioxidant enzymes, immunity, gut microbiota

## Abstract

This study aimed to evaluate the effects of dietary supplementation with sodium butyrate (SB) in different forms on the growth performance, antioxidant capacity, immune response, and intestinal health of largemouth bass (*Micropterus salmoides*). Five diets were formulated: a basal diet (SB0), diets with 1000 (ESB1), 1500 (ESB2), and 2000 mg/kg encapsulated SB (ESB3), and a diet with 2000 mg/kg raw powder sodium butyrate (RSB, non-encapsulated). After 49 days of feeding trials, the ESB2 group exhibited significantly higher weight gain and specific growth rates and a lower feed coefficient than those of the SB0 group (*p* < 0.05). Compared with the SB0 group, proximal intestinal villus length and width were significantly increased in the ESB1, ESB2, and ESB3 groups (*p* < 0.05). The expressions of tight junction genes *zo-1*, *claudin-1*, and *claudin-4* were up-regulated in these SB-supplemented groups and most pronounced in the ESB2 group (*p* < 0.05). Compared with the SB0 group, antioxidant enzyme activities (catalase and superoxide dismutase) and their gene expressions increased in the ESB1, ESB2, and RSB groups (*p* < 0.05). Immune-related genes *il-10* and *tgf-β1* were up-regulated in the ESB1 and ESB2 groups, while their *il-8*, *il-1β*, and *tnf-α* were down-regulated (*p* < 0.05). The ESB2 group had higher intestinal abundance of Firmicutes and *Lactobacillus*. In conclusion, dietary supplementation with 1500 mg/kg encapsulated SB (ESB2) improved growth, antioxidant capacity, immunity, and gut health in largemouth bass.

## 1. Introduction

With the rapid development of aquaculture and marine fishing industries, the demand for fishmeal as a high-quality protein source has continued to rise, and its insufficient supply has caused prices to soar [1]. Plant protein materials, due to their wide availability and low cost, are commonly used as alternatives to fishmeal in aquafeeds. However, anti-nutritional factors and amino acid imbalances in plant protein materials can affect the growth and health of aquatic animals to varying degrees [2,3]. When fishmeal is largely replaced by plant protein, functional feed additives (such as organic acids, nucleotides, and probiotics) are typically added to mitigate the negative effects of the alternative protein sources, thereby enhancing fish growth performance, boosting immunity and disease resistance, and improving gut health [4,5].

Sodium butyrate (SB), the sodium salt of the short-chain volatile fatty acid butyric acid, is widely used in poultry and aquaculture feed due to its high stability, rapid absorption, and environmental friendliness [6,7]. However, different forms of SB show significant differences in bioavailability and efficacy [8]. Raw SB powder, with its strong odor and short shelf life, is rapidly absorbed in the stomach, making it difficult to exert sustained effects in the intestine [9]. Encapsulated SB (ESB), encapsulated with lipids or polymers, forms protective and controlled-release layers. It is released gradually in the stomach and intestinal regions [10] and is targeted to the cecum and colon [11], enhancing intestinal digestion and absorption and reducing inflammation [12,13].

SB is an important energy source for intestinal epithelial cells, promoting cell growth and metabolism [14], and can reduce intestinal pH to suppress harmful bacteria and maintain gut homeostasis [15]. Studies have demonstrated that dietary SB can enhance the growth performance and digestive enzyme activities of juvenile thin-lipped mullet (*Liza ramada*) [16]. Moreover, dietary SB has been proven to enhance intestinal absorption and immunity in various fish species, including Nile tilapia (*Oreochromis niloticus*) [17], grass carp (*Ctenopharyngodon idella*) [18], turbot (*Scophthalmus maximusa*) [19], and rice field eel (*Monopterus albus*) [20]. Interestingly, Jesus et al. [21] observed that the addition of encapsulated SB at 0.5% in either buffer or oil improved the yield and biomass gain compared with the unprotected forms during sexual reversion of Nile tilapia. Additionally, Zhou et al. [22] found that different SB forms improved *C. idella* health by up-regulating *myd88* and *tlr22* gene expressions, enriching beneficial gut bacteria, and reducing lipid accumulation, despite showing no significant effects on growth. These findings highlight the need for further investigation into the optimal dietary supplementation levels of different forms of SB (raw powder or encapsulated), as well as their impacts on gut antioxidant status, microbiota composition, and immune function.

The largemouth bass (*Micropterus salmoides*), a carnivorous fish, preys on small fish and insects. It is highly regarded in the market for its firm flesh, delicious taste, few bones, and high nutritional value [23]. With its rapid growth and adaptability to intensive farming, it has a robust market demand. Largemouth bass has a high demand for dietary protein and is more sensitive to adverse intestinal effects caused by plant-based feed, making it a key species for enhancing feed efficiency and health management and an ideal model for studying carnivorous fish intestinal physiology [24]. Research showed that dietary supplementation with SB in feed could alleviate the negative influence of high-fat diets on fish growth, intestinal microbiota homeostasis, and liver health in largemouth bass [23,24]. However, the appropriate inclusion level of encapsulated SB in largemouth bass feed, along with comparisons to raw powder SB at equivalent doses, remains an area for further investigation. This study investigates how different doses of encapsulated SB and raw powder SB affect the growth, serum biochemistry, gut structure, antioxidant capacity, immunity, and gut microbiota of largemouth bass, aiming to guide the optimal use of encapsulated SB in feed.

## 2. Materials and Methods

### 2.1. Diet Formulation

Largemouth bass (*M. salmoides*) with an initial weight of 29.75 ± 0.25 g were used in this study. Sodium butyrate (SB) as the feed additive was obtained from Hunan Perfly Biotech Co., Ltd. (Hunan, China). Five experimental diets were formulated: a control diet (SB0, basal diet containing 25% fish meal by weight with no SB) and four treatment diets supplemented with 1000 mg/kg (ESB1), 1500 mg/kg (ESB2), and 2000 mg/kg (ESB3) of encapsulated SB, or 2000 mg/kg of raw powder SB (RSB). The addition concentration of SB in the feed referenced effective levels reported in published studies on grass carp (*C. idella*) [22], yellow drum (*Nibea albiflora*) [13], and Nile tilapia [21], with a gradient design covering 1000–2000 mg/kg to identify optimal concentrations for largemouth bass. The main ingredients and nutritional compositions of the five diets are listed in Table 1.

The feed ingredients were finely ground through a 60-mesh sieve, uniformly mixed according to the formulated ratios, and extruded into strip-shaped pellets using an aquafeed extruder (Jiangsu Zhengchang Grain and Feed Machinery Co., Ltd., SZLH200, Liyang, China) at a temperature of 50 °C. The extruded feed was dried to a moisture content of less than 10% and then formed into approximately 3 mm diameter particles using a feed pellet mill (Jiangsu Zhengchang Grain and Feed Machinery Co., Ltd., 1208 Pellet Mill, Liyang, China) for low-temperature storage until use.

### 2.2. Experimental Animals and Management

After two weeks of acclimation, 600 largemouth bass with an initial weight of 29.75 ± 0.25 g were evenly distributed into five groups (n = 120 fish per group). Each group was allocated three net cages (2 m × 2 m × 1.6 m; total 15 cages) with 40 fish per cage. The cages were suspended in an aerated pond. The 49-day feeding trial involved daily feeding at 3% of body weight, distributed twice daily (07:00 and 17:00) until apparent satiation (1–1.5 h per feeding). Weekly, four fish per group were randomly weighed to adjust feeding rations. Water quality parameters were maintained within the following ranges: dissolved oxygen at 5–7 mg/L, water temperature at 16–32 °C, pH at 7.0–8.5, and pond water transparency at 30–40 cm.

### 2.3. Sample Collection and Ethics Statement

At the end of the 49-day rearing period, the largemouth bass were subjected to a 24 h fasting period. Subsequently, 20 fish were randomly sampled from each net cage (60 fish per group) for experimentation. The fish were anesthetized with an MS-222 solution (50 mg/L, Changsha Shanghe Biotechnology Co., Ltd., Changsha, China) for 5 min. All experiments complied with the Guidelines for the Care and Use of Laboratory Animals in China and adhered to local animal welfare regulations and institutional guidelines.

Surface moisture was gently removed from the 60 anesthetized fish per group using sterile gauze before body weight measurement. Blood was collected from the caudal vein of 30 randomly selected fish per group. The blood samples were left to stand at 4 °C for 24 h and then centrifuged at 3000× *g* for 15 min to collect the serum, which was stored at −80 °C for subsequent serum biochemical analysis. The intestines of these 30 fish were quickly dissected on ice, and tissue samples from the proximal (3 cm posterior to the stomach), middle, and distal (3 cm anterior to the anus) segments of the intestine were collected. Tissue samples from six fish were fixed in 4% paraformaldehyde at 4 °C for histological section preparation, while samples from the remaining 24 fish per experimental group (8 fish from each of the 3 tanks) were snap-frozen in liquid nitrogen for 12 h and then stored at −80 °C for total RNA extraction and antioxidant enzyme activity assays.

Finally, from the remaining 30 anesthetized fish in each group, 18 fish per experimental group (6 fish from each of the 3 tanks) were selected for gut content collection. The entire intestinal contents of these fish per experimental group (4 fish from each of the 3 tanks) were collected aseptically and stored in sterile vials. The samples were snap-frozen in liquid nitrogen for 12 h and then stored at −80 °C for subsequent gut microbiota sequencing. The remaining 12 fish per experimental group (4 fish from each of the 3 tanks) were stored at −20 °C for whole-body composition analysis (moisture, crude fat, crude protein, and crude ash).

### 2.4. Survival and Growth Performance

The survival and growth parameters measured in this study included initial body weight (IBW), final body weight (FBW), weight gain rate (WGR), specific growth rate (SGR), Feed coefficient (FC), feed intake (FI), viscera somatic index (VSI), and hepatosomatic index (HSI).

Weight gain rate, WGR (%) = 100 × [(final body weight − initial body weight)/initial body weight].(1)

Specific growth rate, SGR (%/d) = (ln final body weight − ln initial body weight)/days × 100.(2)

Feed intake, FI (g) = total amount of feed consumption (g)/[(initial body weight + final body weight)/2]/days.(3)

Feed coefficient, FC = total amount of feed consumption (g)/(final body weight + death body weight (g) − initial body weight (g)).(4)

Viscerosomatic index, VSI (%) = 100 × visceral weight (g)/body weight (g).(5)

Hepatosomatic index, HSI (%) = 100 × liver weight (g)/body weight (g).(6)

### 2.5. Proximate Composition Analysis

The crude protein content was determined using the Kjeldahl method (N × 6.25). Crude fat content was measured by Soxhlet extraction. Moisture content was determined by drying samples to constant weight in an oven at 105 °C. Ash content was analyzed by incinerating samples to constant weight in a muffle furnace at 550 °C. All procedures followed the methods described by Su et al. [25].

### 2.6. Histological Observation

Proximal, middle, and distal intestinal samples from each group (n = 3) were dehydrated through a graded ethanol series and embedded in paraffin. Transverse sections approximately 1 cm in length were cut into 5 μm thick slices and stained with hematoxylin and eosin (H&E) following the method of Su et al. [25]. H&E staining was performed and scanned by Wuhan Sevicebio Technology Co., Ltd. (Wuhan, China). Sections were scanned using a NanoZoomer S60 (Hamamatsu Photonics, Hamamatsu, Japan), and measurements of villus length, villus width, and muscle layer thickness were taken using Image-Pro Plus 6.0 software.

### 2.7. Serum Biochemical Assays

Blood samples collected from the caudal vein were left to clot at 4 °C, then centrifuged at 3000× *g* for 10 min to obtain serum, which was stored at −80 °C. Serum samples (n = 3) were analyzed for alanine aminotransferase (ALT), aspartate aminotransferase (AST), albumin (ALB), glucose (GLU), and total protein (TP) using a fully automatic biochemical analyzer (Mindray, BS280, Shenzhen, China) [26].

### 2.8. Intestinal Antioxidant Enzyme Activity Assays

Antioxidant enzyme activities in the proximal, middle, and distal intestines of largemouth bass were measured using commercial kits from Nanjing Jiancheng Bioengineering Institute (Nanjing, China). Superoxide dismutase (SOD) and catalase (CAT) activities and the total protein content (TP) were determined following the manufacturer’s protocols [25,26].

### 2.9. Real-Time Quantitative PCR Analysis

Total RNA was extracted from the proximal, middle, and distal intestine of each group of largemouth bass using the RNAex Pro Reagent (Hunan Akrie Biotechnology Co., Ltd., Changsha, China) and quantified using a NanoDrop 2000 (Thermo Scientific, Waltham, MA, USA). cDNA was synthesized from total RNA using the gDNA Clean Reaction Mix Ver.2 and 5X Evo M-MLV RT Reaction Mix II (Yeasen Biotechnology (Shanghai) Co., Ltd., Shanghai, China) and stored at −20 °C. Primers for real-time quantitative PCR (RT-qPCR) were designed using Primer Premier 5.0 and synthesized by Sangon Biotech (Shanghai) Co., Ltd. (Shanghai, China) (Table 2). RT-qPCR was performed using the CFX 96TM Real-Time System (Bio-Rad, Hercules, CA, USA). The total reaction volume was 20 μL, including 10 μL of SYBR Green, 0.4 μL of upstream and downstream specific primers (10 mM each), 1 μL of template cDNA, and 8.2 μL of sterile deionized water. The reaction protocol included an initial denaturation at 95 °C for 30 s, followed by 40 cycles of 95 °C for 5 s, annealing at 60 °C for 30 s, and a final melting curve analysis from 65 °C to 95 °C with a 5 s hold every 0.5 °C. β-actin was used as the reference gene, and relative gene expression was measured using the 2^−△△Ct^ method [26,27].

### 2.10. Gut Microbiota Analysis

Total intestinal genomic DNA was extracted using the E.Z.N.A.^®^ Soil DNA Kit (Omega, Irving, TX, USA). The V3+V4 region of 16S rRNA was amplified with primers 338F (5′-ACTCCTACGGGAGGCAGCA-3′) and 806R (5′-GGACTACHVGGGTWTCTAAT-3′). Sequencing was performed on the Illumina MiSeq platform (Shanghai Majorbio Biopharm Technology Co., Ltd., Shanghai, China). Raw data were merged using FLASH (v1.2.7) and filtered for quality with QIIME2 (v2020.2), followed by chimera removal with UCHIME (v8.1) to obtain high-quality tags. Effective sequences were analyzed using USEARCH 10.0 for OTU clustering at 97% similarity. Taxonomic annotation of OTUs was performed with the RDP Classifier 2.2 against the Silva 16S rRNA gene database (v132, http://www.arb-silva.de accessed on 11 April 2025). Microbial community composition was profiled at various taxonomic levels (phylum, class, order, family, genus, species), and diversity indices (Shannon, Simpson, Chao, Ace) were calculated. β-diversity was assessed via principal coordinate analysis (PCoA, Vegan v2.4.3), and differentially abundant biomarkers were identified using linear discriminant analysis effect size (LEfSe) [28].

### 2.11. Data Analysis

Data are expressed as the mean ± standard error (mean ± S.E.) (n = 3). Statistical analyses were performed using IBM SPSS Statistics 26.0, and graphs were generated with GraphPad Prism 9.0. Prior to analysis, data that deviated from the population mean were identified and excluded through a one-sample *t*-test (α = 0.05). One-way ANOVA followed by Duncan’s multiple range test was used for intergroup comparisons. Paired-samples *t*-tests were used for intragroup comparisons of gut locations. Significance was set at *p* < 0.05. Significant differences among different gut locations within the same SB group are indicated by different capital letters, while significant differences among different SB groups within the same gut location are indicated by different lowercase letters.

## 3. Results

### 3.1. Growth Performance and Body Composition Analysis

As shown in Table 3, the WGR (303.53 ± 17.92%) and SGR (2.41 ± 0.13%/d) of the ESB2 group were significantly higher than those of the SB0 group (261.95 ± 1.39% and 2.10 ± 0.01%/d, respectively) (*p* < 0.05). Meanwhile, the FC (0.93 ± 0.09) of the ESB2 group was significantly lower than that of the SB0 group (1.20 ± 0.04) (*p* < 0.05). However, no significant differences were observed in these parameters among the remaining four groups (*p* > 0.05).

From Table 4, it is evident that there were no significant differences in moisture, crude protein, crude fat, and crude ash content among the different groups (*p* > 0.05).

### 3.2. Intestinal Histomorphology

**Figure 1:**
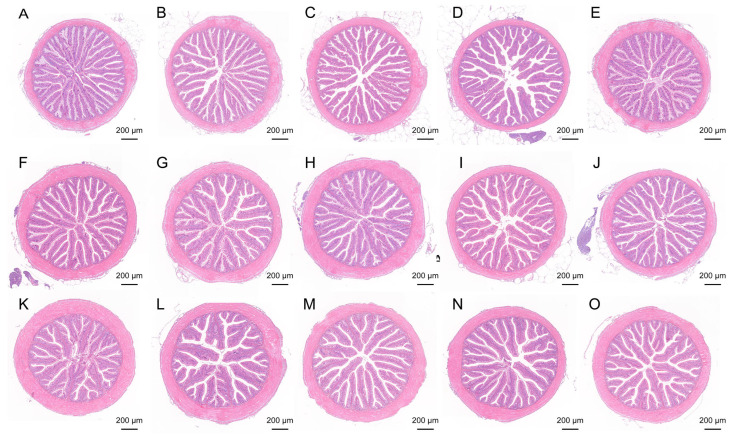
Histological HE staining results of intestinal tissue in five groups of largemouth bass. (**A**–**E**) HE staining of the proximal intestine; (**F**–**J**) HE staining of the mid intestine; (**K**–**O**) HE staining of the distal intestine.

The H&E staining results of the intestinal tissues (proximal, mid, and distal segments) in largemouth bass are presented in Figure 1. All groups exhibited intact intestinal architecture, orderly arranged villi, and smooth mucosal surfaces across the proximal, mid, and distal regions. In the ESB1, ESB2, and ESB3 groups, both the villus length and width in the proximal intestine were significantly increased compared to the control (*p* < 0.05). Notably, the muscularis thickness of the proximal intestine was significantly elevated in the ESB2 group (*p* < 0.05). Moreover, the villus width and muscularis thickness in the mid and distal intestines displayed a dose-dependent enhancement with increasing SB concentrations across the ESB groups. In the RSB group, the villus length in the distal intestine and villus width in the proximal intestine were significantly higher than those in the SB0 group (*p* < 0.05) (Table 5).

### 3.3. Tight Junction Gene Expression

**Figure 2:**
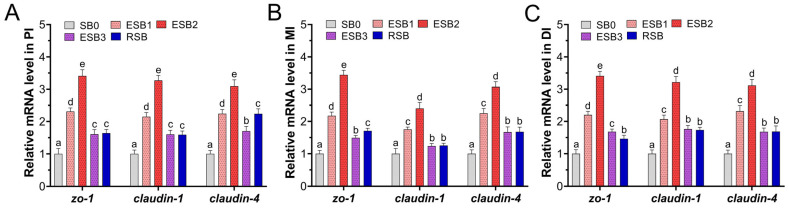
Effects of dietary SB levels on intestinal tight junction gene expression in largemouth bass. (**A**) Expression of tight junction genes in the proximal intestine (PI); (**B**) expression of tight junction genes in the mid intestine (MI); (**C**) expression of tight junction genes in the distal intestine (DI). Different lowercase letters indicate significant differences between groups (*p* < 0.05), while the same lowercase letters indicate no significant differences (*p* > 0.05).

As shown in Figure 2, the expression of tight junction-related genes (*zo-1*, *claudin-1*, and *claudin-4*) in the proximal, middle, and distal intestine was significantly up-regulated in the ESB1, ESB2, ESB3, and RSB groups compared to the SB0 group (*p* < 0.05). Among these, the highest gene expression levels were observed in the ESB2 group, indicating that the intestinal tight junction function was strongest when the diet contained 1500 mg/kg of encapsulated SB.

### 3.4. Serum Biochemical Parameters

In the serum biochemistry parameters of largemouth bass, the levels of GLU, ALB, and AST showed no significant differences among the five groups (*p* > 0.05). The TP content was lowest in the ESB3 group (29.89 ± 2.86 g/L) and was significantly lower than in the SB0 (35.62 ± 4.20 g/L) and ESB2 (35.43 ± 3.26 g/L) groups (*p* < 0.05). The ALT level in the SB0 group (3.65 ± 1.51 U/L) was significantly lower than in the RSB group (5.49 ± 1.39 U/L) (*p* < 0.05), but no significant differences were found between the SB0 group and the ESB1, ESB2, or ESB3 groups (*p* > 0.05) (Table 6).

### 3.5. Intestinal Antioxidant Capacity

**Figure 3:**
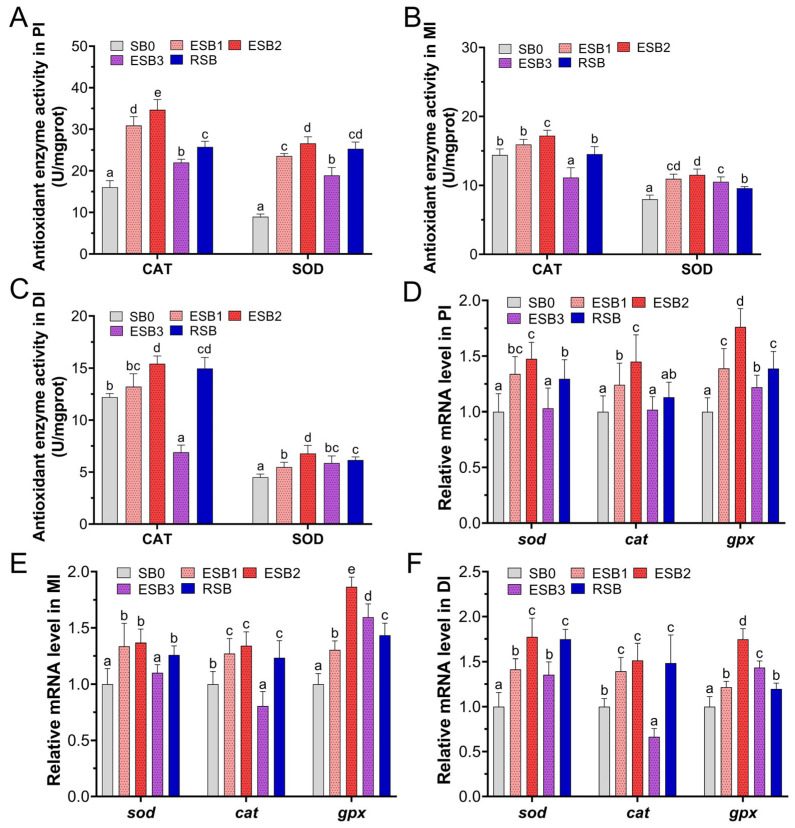
Effects of dietary SB levels on intestinal antioxidant enzyme activities and antioxidant gene expression in largemouth bass. (**A**) CAT and SOD enzyme activities in the proximal intestine (PI); (**B**) CAT and SOD enzyme activities in the mid intestine (MI); (**C**) CAT and SOD enzyme activities in the distal intestine (DI); (**D**) antioxidant gene expression in the proximal intestine (PI); (**E**) antioxidant gene expression in the mid intestine (MI); (**F**) antioxidant gene expression in the distal intestine (DI). Different lowercase letters indicate significant differences between groups (*p* < 0.05), while the same lowercase letters indicate no significant differences (*p* > 0.05).

As shown in Figure 3, the activity trends of the antioxidant enzymes SOD and CAT in the intestines of largemouth bass were similar across the five groups, with a stepwise decrease in enzyme activity from the proximal to middle and distal intestines. Compared to the SB0 group, the SOD activity in the proximal, middle, and distal intestine was significantly increased in the ESB1, ESB2, ESB3, and RSB groups (*p* < 0.05) (Figure 3A–C). In the proximal intestine, the CAT activity in the ESB1, ESB2, ESB3, and RSB groups was significantly higher than in the SB0 group (*p* < 0.05) (Figure 3A). In the middle intestine, only the CAT activity in the ESB3 group was significantly lower than in the SB0 and the other three groups (*p* < 0.05) (Figure 3B). In the distal intestine, the CAT activity in the ESB2 and RSB groups was significantly higher than in the SB0 group (*p* < 0.05), while the CAT activity in the ESB3 group was significantly lower than in the SB0 group (*p* < 0.05) (Figure 3C). Overall, the ESB2 group exhibited the highest enzyme activity for both CAT and SOD across the proximal, middle, and distal intestines, indicating that dietary supplementation with 1500 mg/kg encapsulated SB enhanced antioxidant capacity the most.

At the gene expression level, in the proximal and middle intestines, the expression of the *sod* gene was significantly up-regulated in the ESB1, ESB2, and RSB groups compared to the SB0 group (*p* < 0.05) (Figure 3D,E). In the distal intestine, *sod* gene expression was significantly higher in the four SB-supplemented groups than in the SB0 group (*p* < 0.05) (Figure 3F). In the proximal intestine, *cat* gene expression was significantly higher in the ESB1 and ESB2 groups than in the SB0 group (*p* < 0.05) (Figure 3D). In the middle and distal intestine, *cat* gene expression was significantly higher in the ESB1, ESB2, and RSB groups than in the SB0 group (*p* < 0.05), while the ESB3 group showed significantly lower *cat* gene expression than the SB0 group (*p* < 0.05) (Figure 3E,F). The expression of the *gpx* gene was significantly higher in the four SB-supplemented groups than in the SB0 group across the proximal, middle, and distal intestine (*p* < 0.05) (Figure 3D–F).

### 3.6. Intestinal Immunity

**Figure 4:**
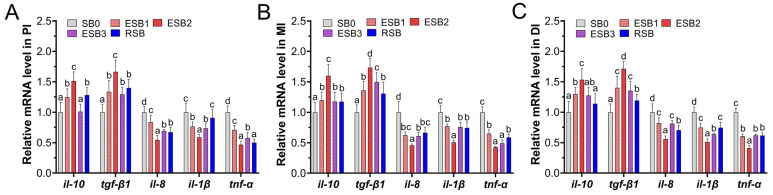
Effects of dietary SB levels on intestinal immune gene expression in largemouth bass. (**A**) Immune gene expression in the proximal intestine (PI); (**B**) immune gene expression in the mid intestine (MI); (**C**) immune gene expression in the distal intestine (DI). Different lowercase letters indicate significant differences between groups (*p* < 0.05), while the same lowercase letters indicate no significant differences (*p* > 0.05).

As shown in Figure 4, the expression of the immune-related genes *tgf-β1* and *il-10* in the proximal intestine of largemouth bass was significantly higher in the ESB1, ESB2, and RSB groups than in the SB0 group (*p* < 0.05) (Figure 4A). In the middle intestine, the expression of these two genes was significantly higher in all four groups compared to the SB0 group (*p* < 0.05) (Figure 4B). In the distal intestine, the expression of *tgf-β1* and *il-10* genes was significantly higher in the ESB1 and ESB2 groups than in the SB0 group (*p* < 0.05) (Figure 4C). The expression of the *il-1β* gene in the RSB group was significantly higher than in the SB0 group in the middle and distal intestine (*p* < 0.05) (Figure 4B,C), but no significant difference was found in the proximal intestine (*p* > 0.05) (Figure 4A). The expression of *il-8* and *tnf-α* genes was significantly higher in the ESB1, ESB2, ESB3, and RSB groups than in the SB0 group across all intestinal segments (Figure 4A–C) (*p* < 0.05).

From the above analysis, the expression levels of the anti-inflammatory genes *tgf-β1* and *il-10* in the ESB2 group were higher than in the other groups across the proximal, middle, and distal intestines (*p* < 0.05). In contrast, the expression levels of the pro-inflammatory genes *il-1β*, *il-8*, and *tnf-α* in the ESB2 group were lower than in the other groups. Thus, dietary supplementation with 1500 mg/kg encapsulated SB enhances the anti-inflammatory ability of largemouth bass and reduce the incidence of inflammation in the intestine.

### 3.7. Intestinal Microbiota and Immune Gene Correlation

As shown in Table 7, the microbial diversity indices (Sobs, Chao, Ace, and Shannon) were significantly higher in the SB0 group than in the RSB group (*p* < 0.05), while no significant differences were found between the three encapsulated SB groups (ESB1, ESB2, ESB3) and the SB0 or RSB groups (*p* > 0.05). The Simpson index was significantly lower in the SB0 group than in the RSB group (*p* < 0.05), with no significant differences among the ESB groups (*p* > 0.05).

**Figure 5:**
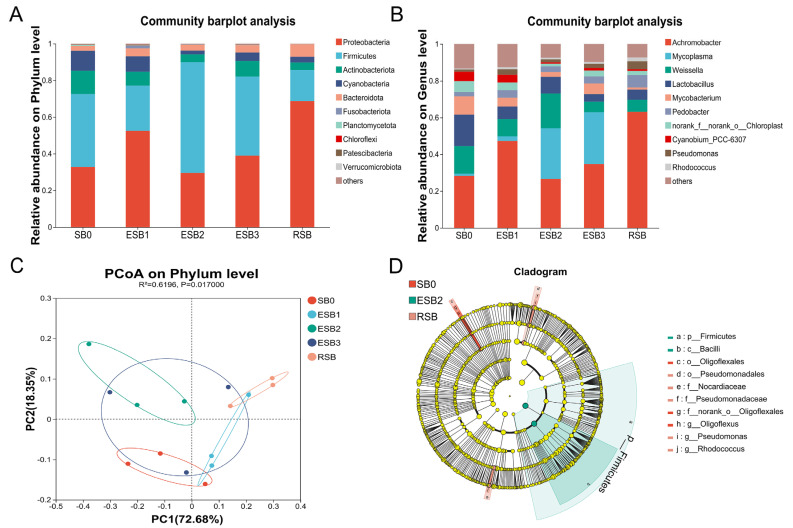
Changes in the intestinal microbiota composition in different SB groups. (**A**) Intestinal microbiota composition analysis at the phylum level; (**B**) intestinal microbiota composition analysis at the genus level; (**C**) principal coordinate analysis (PCoA); (**D**) LEfse analysis of intestinal microbiota from phylum to genus.

At the phylum level, the dominant phyla in the gut microbiota of largemouth bass were Firmicutes, Proteobacteria, Actinobacteria, and Cyanobacteria. The abundance of Firmicutes was significantly higher in the ESB2 group than in the SB0 group (*p* < 0.05), while the abundance of Proteobacteria and Actinobacteria showed a decreasing trend but without significant differences (*p* > 0.05) (Figure 5A). At the genus level, the dominant genera across all groups were *Achromobacter*, *Mycoplasma*, *Weissella*, and *Lactobacillus*. The abundances of *Weissella* and *Lactobacillus* were significantly higher in the ESB2 group than in the SB0 group (*p* < 0.05) (Figure 5B). Principal coordinate analysis (PCoA) revealed distinct differences in gut microbiota composition between the SB-supplemented groups and the SB0 group at the phylum level (Figure 5C).

LEfse analysis (Figure 5D) indicated that the OTU abundance of beneficial bacteria, including Firmicutes and Bacilli, was significantly higher in the ESB2 group than in the other groups (*p* < 0.05). In contrast, the OTU abundance of Pseudomonadales, Nocardiaceae, Pseudomonadaceae, *Pseudomonas*, and *Rhodococcus* was significantly higher in the RSB group (*p* < 0.05). The OTU abundance of Oligoflexales and *Oligoflexus* was significantly higher in the SB0 group than in the other groups (*p* < 0.05).

**Figure 6:**
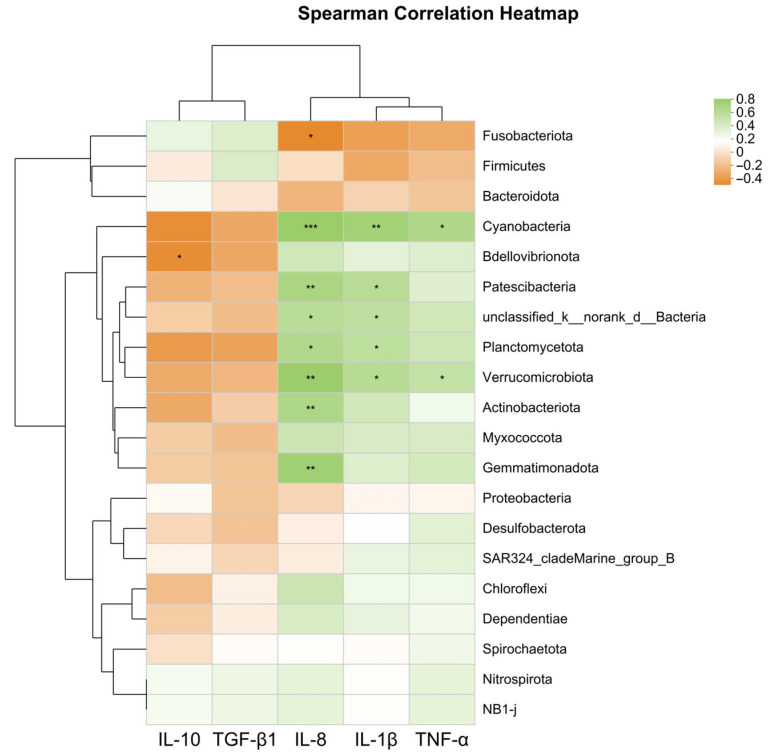
Heatmap correlation analysis of intestinal microbiota at the phylum level and immune gene expression levels affected by SB. * Indicates *p* < 0.05, ** indicates *p* < 0.01, *** indicates *p* < 0.001.

As shown in Figure 6, the expression level of the anti-inflammatory gene *il-10* was significantly negatively correlated with Bdellovibrionota (*p* < 0.05). The expression levels of pro-inflammatory genes *tnf-α*, *il-1β*, and *il-8* were significantly positively correlated with Planctomycetota, Verrucomicrobiota, Cyanobacteria, Actinobacteriota, and Gemmatimonadota (*p* < 0.05).

## 4. Discussion

### 4.1. Effects of SB Supplementation on Growth Performance of Largemouth Bass

As a novel green feed additive, SB has been widely applied in aquatic diets to enhance growth performance. In herbivorous species, dietary supplementation with 300 mg/kg SB improved weight gain rate (WGR) and feed coefficient (FC) in common carp (*Cyprinus carpio*) [29]. Similarly, 1000–2000 mg/kg SB significantly elevated the specific growth rate (SGR) of grass carp (*C*. *idellus*) [30]. For omnivorous fish, 300 mg/kg SB increased WGR, SGR, and FC in Nile tilapia (*O*. *niloticus*) [31,32], while 2000–4000 mg/kg SB enhanced growth performance in crucian carp (*Carassius auratus*) [33]. In carnivorous species, 3000 mg/kg palm oil-encapsulated SB improved WGR, SGR, and FC in gilthead sea bream (*Sparus aurata*) [34]. However, no significant growth benefits were observed in Atlantic salmon (*Salmo salar*) or rainbow trout (*Oncorhynchus mykiss*) fed 5000–20,000 mg/kg SB [35,36].

Notably, Abdel-Mohsen et al. [37] reported that 0.2% SB promoted growth in European sea bass (*Dicentrarchus labrax*) juveniles, whereas 0.3% SB showed no significant effects. A recent study by Hou et al. [38] demonstrated that 2000 mg/kg SB significantly enhanced growth in largemouth bass. In the present study, dietary supplementation with 1500 mg/kg encapsulated SB (ESB2) significantly increased WGR and SGR while reducing FC in largemouth bass (*p* < 0.05). In contrast, neither lower (1000 mg/kg) nor higher (2000 mg/kg) encapsulated SB doses nor 2000 mg/kg raw powder SB improved these parameters. These discrepancies may stem from dose-dependent efficacy, formulation differences (encapsulated vs. raw powder), and species-specific responses influenced by feeding habits and metabolic pathways.

### 4.2. Effects of SB Addition on Intestinal Morphology and Tight Junction Capacity in Largemouth Bass

Intestinal morphological parameters, such as villus length, crypt depth, and muscle layer thickness, are key biomarkers for evaluating the digestive and absorptive efficiency of fish [39]. In this study, the addition of encapsulated SB (1000–2000 mg/kg) to feed significantly increased the villus length and width in the proximal, mid, and distal regions of the intestine of largemouth bass, with a positive correlation observed as the concentration of SB increased. These results are consistent with findings in Nile tilapia (*O*. *niloticus*) fry [40] and grass carp (*C*. *idellus*) [22]. In this study, largemouth bass fed with feed containing 2000 mg/kg raw powder SB showed a significant increase in villus length only in the distal intestine and a significant increase in villus width only in the proximal intestine compared to the control group. This suggests that the effect of 2000 mg/kg raw powder SB on improving intestinal villus structure in largemouth bass is inferior to that of encapsulated SB, indicating that the sustained-release technology of encapsulated SB facilitates its gradual release throughout the intestine, thereby enhancing digestive and absorptive capacity through improved villus structure.

**Figure 7:**
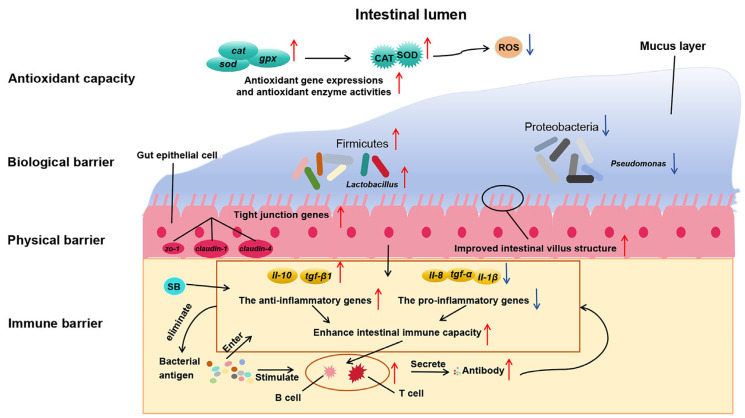
Schematic of SB action on the intestine of largemouth bass. The red upward arrow indicates an increase in the enzyme activities, gene expressions or abundance of gut microbiota, while the blue downward arrow indicates a decrease in the enzyme activities, gene expressions or abundance of gut microbiota.

The physical barrier function of fish intestines maintains the stability of intestinal epithelial cells and their interconnections, ensuring normal function and integrity [41]. Tight junction proteins, such as Claudins, Occludin, and ZOs, play a critical role in limiting macromolecular passage and regulating cellular barrier permeability [42,43]. Studies have shown that adding 2000 mg/kg SB to a high-soybean-meal diet (37.9%) for turbot (*S*. *maximus*) significantly up-regulated the expression of *zo-1*, *claudin-4*, and *occludin* genes, which were previously down-regulated [19]. In this study, the addition of 1000–2000 mg/kg encapsulated SB and 2000 mg/kg raw powder SB to a basal diet (containing 25% fishmeal) significantly up-regulated the gene expression of *zo-1*, *claudin-1*, and *claudin-4* in the intestines of largemouth bass. In Nile tilapia (*O. niloticus*), adding 2–4 g/kg SB to the 60 % fava bean feed showed that the structural integrity of the intestinal barrier was associated with improved muscle texture, because genes associated with intestinal tight junctions (*zo-1* and *claudin-14*) and the anti-apoptotic gene *bcl2* showed significant positive correlations with muscle texture parameters (hardness, chewiness, gumminess, springiness, adhesiveness, resilience, and cohesiveness) [44]. In summary, both encapsulated SB and raw powder SB can improve intestinal morphology, promote tight junctions in intestinal epithelial cells, and enhance the physical barrier function of the intestine in largemouth bass (Figure 7).

### 4.3. Effects of Sodium Butyrate Supplementation on Serum Biochemical Parameters of Largemouth Bass

Serum biochemical parameters serve as critical indicators of physiological status and overall health in fish, providing essential diagnostic tools for disease detection [45,46]. Aspartate aminotransferase (AST) and alanine aminotransferase (ALT) are sensitive biomarkers for evaluating hepatopancreatic function and cellular integrity [47]. In the present study, AST levels showed no significant differences among groups (*p* > 0.05), indicating neither encapsulated nor non-encapsulated SB induced substantial hepatocyte or myocardial injury. ALT was significantly elevated in the RSB group versus SB0 (*p* < 0.05), while encapsulated SB groups (ESB1-ESB3) remained comparable to controls, potentially attributable to rapid gastric release of non-encapsulated SB. Contrastingly, studies in crucian carp (*C. auratus*) reported decreased AST and ALT following 1000–2000 mg/kg SB supplementation [48], which may be explained by differences in fish species and feed formulations. Total protein (TP), a core indicator of immune function and nutritional metabolism [49], significantly decreased in the ESB3 group relative to SB0 and ESB2 (*p* < 0.05), suggesting that a high concentration of encapsulated SB (2000 mg/kg) may inhibit hepatic protein synthesis in largemouth bass.

### 4.4. Effects of SB Addition on Intestinal Antioxidant Capacity in Largemouth Bass

Antioxidant capacity is a core indicator for evaluating the health status and environmental adaptability of aquatic animals [50]. Superoxide dismutase (SOD) and catalase (CAT), as key antioxidant enzymes in fish, play an important role in combating oxidative stress damage [51]. In this study, RT-qPCR analysis showed that both encapsulated SB (1000–2000 mg/kg) and raw powder SB (2000 mg/kg) significantly up-regulated the mRNA expression of *sod*, *cat*, and *gpx* genes in the intestine of largemouth bass, which is consistent with previous findings in largemouth bass [52]. Results of antioxidant enzyme activity demonstrated that the addition of encapsulated SB and raw powder SB to feed significantly enhanced SOD activity in the proximal, mid, and distal regions of the intestine of largemouth bass. These results align with previous studies on the antioxidant effects of SB in other fish species. For example, adding 500 mg/kg SB to the feed of rice field eel (*M*. *albus*) and grass carp (*C*. *idellus*) improved SOD and GSH-Px activities in the hepatopancreas and intestine [20,22]. Similarly, adding 0.05–0.2% raw powder SB to the feed of largemouth bass significantly increased the activity levels of T-SOD, CAT, and GPx [23,24].

Notably, in this study, the CAT activity in the group fed with 2000 mg/kg encapsulated SB was significantly lower than that in the group fed with 2000 mg/kg raw powder SB and in groups fed with low to medium concentrations (1000–1500 mg/kg) of encapsulated SB. Additionally, the SOD and CAT activities in the intestine of largemouth bass fed with encapsulated SB and raw powder SB showed a gradual decline in the proximal, mid, and distal regions of the intestine. This suggests that low concentrations of SB can enhance antioxidant capacity in fish, but its gradual absorption in the intestine leads to a weakening of its antioxidant effects. However, high concentrations (2000 mg/kg) of encapsulated SB may cause oxidative stress imbalance, disrupt intracellular redox homeostasis, and lead to excessive reactive oxygen species (ROS) production, exceeding the clearance capacity of antioxidant enzymes, thereby reducing CAT activity [53]. This study found that adding 1500 mg/kg encapsulated SB provided the strongest antioxidant capacity in fish, while adding 2000 mg/kg encapsulated SB may promote intestinal stress responses (Figure 7). This indicates that higher concentrations of SB in feed are not necessarily better. Furthermore, the addition of raw powder SB should be appropriately higher than that of encapsulated SB to achieve equivalent effects in the intestine.

### 4.5. Effects of SB Addition on Intestinal Immune Capacity in Largemouth Bass

Cytokines play a crucial role in fish immune health. Pro-inflammatory cytokines such as TNF-α, IL-1β, and IL-8 are involved in immune responses, while anti-inflammatory cytokines such as TGF-β1 and IL-10 help modulate inflammation [54]. In this study, the addition of 1000 and 1500 mg/kg encapsulated SB to feed significantly up-regulated the expression levels of anti-inflammatory cytokine genes *il-10* and *tgf-β1* in the proximal, mid, and distal regions of the intestine of largemouth bass, while down-regulating the expression levels of pro-inflammatory cytokine genes *il-8*, *il-1β*, and *tnf-α*. Similar results were observed in common carp (*C*. *carpio*), where supplementing 300 mg/kg SB alleviated soybean oil-induced enteritis by suppressing *il-1β* and *tnf-α* gene expressions and promoting *tgf-β* gene expression [19]. In yellow drum (*N. albiflora*, Richardson), adding 0.15% SB to a high-soybean-meal diet reduced the gene expressions of *tnf-α*, *il-1β*, and *il-6* while significantly increasing *tgf-β1* and *il-10* gene expressions [13]. In largemouth bass fed with high-soybean-meal (28.6%) or high-fat (17.80%) diets, adding 0.2% raw powder SB significantly reduced *tnf-α* and *il-1β* gene expressions, effectively mitigating intestinal inflammation caused by these diets [54,55].

However, this study found that adding high concentrations (2000 mg/kg) of encapsulated SB or raw powder SB to feed reduced the gene expression levels of *il-8*, *il-1β*, and *tnf-α* in the intestine and increased *tgf-β1* gene expression in the distal intestine but showed no significant changes in *il-10* gene expression. In European sea bass (*D*. *labrax*), feeding a soybean diet containing 0.2% SB significantly increased *tnf-α* gene expression while showing no significant changes in *il-1β*, *il-8*, or *il-10* gene expressions [56], likely due to interactions between anti-nutritional factors in soybean meal and SB. Ge et al. [57] found that adding 2000 and 20,000 mg/kg raw powder SB to feed increased *tnf-α* and *il-1β* gene expressions in the liver of largemouth bass, with *il-1β* gene expression increasing with SB concentrations while reducing *tnf-α* gene expression in the intestine. This suggests that high concentrations of SB may affect liver immune function. In summary, adding 1500 mg/kg encapsulated SB to feed promotes the gene expression of anti-inflammatory factors and reduces pro-inflammatory factors in the proximal, mid, and distal regions of the intestine, thereby enhancing the mucosal immune system and serving as an immune barrier in fish (Figure 7).

### 4.6. Effects of SB Addition on the Intestinal Microbial Community Structure of Largemouth Bass

Intestinal microbiota, as part of the intestinal biological barrier, play a crucial role in maintaining host immune function and resisting pathogens [23]. LEfse analysis showed that adding 1500 mg/kg encapsulated SB significantly increased the abundance of Firmicutes in the intestine of largemouth bass, while the abundance of Cyanobacteria, Actinobacteria, and Proteobacteria showed a downward trend, with no significant effect on Bacteroidetes. Firmicutes can improve gut microbiota structure, regulate host metabolism of nutrients such as fats and carbohydrates, and enhance intestinal digestion and utilization of nutrients [44,58,59]. The decrease in Proteobacteria abundance may be due to the inhibitory effect of 1500 mg/kg encapsulated SB on pathogenic bacteria in the intestine. Chen et al. found that adding 0.2% raw SB to the diet significantly increased the abundance of Firmicutes and Bacteroidetes at the phylum level [23,24]. However, in this study, adding 2000 mg/kg raw powder SB reduced the abundance of Firmicutes and increased that of Proteobacteria. The outer membrane of Proteobacteria is mainly composed of lipopolysaccharides, and an increase in their abundance may induce intestinal inflammation in fish [60]. These differences in microbial changes may be due to variations in feed formulation (e.g., protein source), farming environment (e.g., water temperature, dissolved oxygen), or sample processing methods across different studies. Additionally, adding 2000 mg/kg raw powder SB significantly increased the abundance of *Pseudomonas* and *Rhodococcus*. *Pseudomonas* can produce virulence factors and metabolic products in the fish intestine, release inflammatory factors, directly damage the intestinal mucosal barrier, trigger inflammatory responses, and increase the risk of infection in fish [61]. *Pseudomonas* has a high degree of metabolic diversity and can utilize various carbon sources (e.g., butyrate derivatives). *Rhodococcus* is known for its lipid-degrading ability and may obtain energy by breaking down SB. The increase in the abundance of these two genera may be due to the addition of SB providing them with sufficient carbon sources and energy.

In summary, this study found that adding 1500 mg/kg encapsulated SB to feed significantly increased the abundance of Firmicutes in the intestine of largemouth bass, with a downward trend in the abundance of Proteobacteria and Actinobacteria. This indicates that SB addition promotes the colonization and growth of beneficial bacteria in the intestine of largemouth bass and inhibits the adhesion of harmful bacteria. By regulating the dynamic balance of intestinal microbiota, it enhances the biological barrier function of the intestine in largemouth bass (Figure 7).

### 4.7. Outlook

While this study examined the effects of three encapsulated SB concentrations on largemouth bass growth performance, antioxidant capacity, immunity, and intestinal health—including comparisons between encapsulated and non-encapsulated forms—subsequent research will optimize dosage through 5–7 concentration gradients. Future investigations will evaluate sensory attributes via muscle quality and volatile flavor compound analyses, integrated with carbohydrate, protein, and lipid metabolism studies, employing response surface methodology or nonlinear regression (e.g., quadratic polynomial fitting) to identify optimal inclusion levels under multifactorial interactions.

## 5. Conclusions

In this study, both encapsulated SB and raw powder SB up-regulated the expression of tight junction genes (*zo-1*, *claudin-1*, and *claudin-4*), thereby enhancing the intestinal physical barrier function and improving nutrient absorption efficiency. They also promoted the gene expression of anti-inflammatory factors (*tgf-β1* and *il-10*) and inhibited the gene expression of pro-inflammatory factors (*tnf-α*, *il-1β*, and *il-8*), thereby strengthening the intestinal immune barrier. Moreover, adding 1000 and 1500 mg/kg encapsulated SB enhanced the expression of antioxidant-related genes (*sod*, *cat*, and *gpx*) and enzyme activities (SOD and CAT), alleviating oxidative stress damage and systematically improving the fish’s antioxidant capacity. In this study, adding 1500 mg/kg encapsulated SB improved nutrient absorption efficiency, thereby achieving efficient growth conversion, enhancing the growth performance of largemouth bass, and significantly increasing the abundance of beneficial bacteria (Firmicutes) while reducing the proportion of potential pathogenic bacteria (Proteobacteria), thus maintaining intestinal microbial balance and enhancing the intestinal biological barrier. In summary, adding 1500 mg/kg encapsulated SB (ESB2 group) was the most effective, significantly improving the growth performance, intestinal health, and immune function of largemouth bass. This study provides a theoretical basis for developing efficient and eco-friendly feed additives and reduces the farming cost of largemouth bass.

## Figures and Tables

**Table 1:** Experimental feed formulation and chemical composition (% dry matter).

Ingredients	SB0	ESB1	ESB2	ESB3	RSB
Fish meal	25.00	25.00	25.00	25.00	25.00
Fermented soybean meal	5.00	5.00	5.00	5.00	5.00
Chicken meal	15.00	15.00	15.00	15.00	15.00
Soybean meal	25.00	25.00	25.00	25.00	25.00
Cottonseed protein	5.00	5.00	5.00	5.00	5.00
Wheat protein powder	4.20	4.20	4.20	4.20	4.20
Wheat flour	3.25	3.25	3.25	3.25	3.25
Tapioca flour	5.00	5.00	5.00	5.00	5.00
Rice bran	0.00	0.00	0.00	0.00	0.00
Encapsulated SB	0.00	0.10	0.15	0.20	0.00
Raw powder SB	0.00	0.00	0.00	0.00	0.20
Bentonite	2.00	1.90	1.85	1.80	1.80
Freshwater fish oil	6.30	6.30	6.30	6.30	6.30
Calcium monobasic phosphate	1.50	1.50	1.50	1.50	1.50
Lysine	0.65	0.65	0.65	0.65	0.65
Methionine	0.25	0.25	0.25	0.25	0.25
Mineral premix ^1^	0.50	0.50	0.50	0.50	0.50
Vitamin premix ^2^	0.25	0.25	0.25	0.25	0.25
Vitamin C	0.10	0.10	0.10	0.10	0.10
Choline chloride	0.40	0.40	0.40	0.40	0.40
DMPT (C_5_H_11_SO_2_Br)	0.05	0.05	0.05	0.05	0.05
Grammycta	0.05	0.05	0.05	0.05	0.05
Adhesive	0.50	0.50	0.50	0.50	0.50
Sum	100	100	100	100	100
Nutrient composition					
Moisture (%DM)	7.30	7.43	7.64	7.12	7.34
Crude portion (%DM)	49.48	49.53	49.67	49.28	49.55
Crude lipid (%DM)	10.60	10.63	10.71	10.52	10.61
Ash (%DM)	13.70	13.87	13.98	13.56	13.72

^1^ Mineral premix (per kg of feed): CuSO_4_ 25 mg, FeSO_4_ 407 mg, ZnO 50 mg, MnSO_4_ 36 mg, Na_2_SeO_3_ 1.8 mg, MgSO_4_ 4 g; ^2^ Vitamin premix (per kg of feed): inositol 600 mg, vitamin A 40 mg, vitamin D_3_ 0.06 mg, vitamin E 200 mg, vitamin K_3_ 10 mg, vitamin B_1_ (thiamine) 15 mg, vitamin B_2_ (riboflavin) 25 mg, vitamin B_6_ 20 mg, pantothenic acid 50 mg, vitamin B_3_ (nicotinic acid) 200 mg, biotin 3.2 mg, vitamin B_12_ 0.1 mg, folic acid 10 mg, vitamin C 210 mg.

**Table 2:** RT-qPCR primer sequences for largemouth bass.

Gene	Primer	Primer Sequence (5′-3′)	Annealing Temperature (°C)
*β-actin*	F	GGACACGGAAAGGATTGACAG	60
	R	CGGAGTCTCGTTCGTTATCGG	
*sod*	F	CCACCAGAGGTCTCACAGCA	62.2
	R	CCACTGAACCGAAGAAGGACT	
*cat*	F	TGGTGTTCACGGATGAGATGG	60.8
	R	GGAGAAGCGGACAGCAATAGG	
*gpx*	F	ATACCAAGTCTCCTTCCCTCTGT	59
	R	CGTCCACCACTTTGCCATT	
*il-10*	F	CGGCACAGAAATCCCAGAGC	62.1
	R	CAGCAGGCTCACAAAATAAACATCT	
*tgf-β1*	F	GCTCAAAGAGAGCGAGGATG	59
	R	TCCTCTACCATTCGCAATCC	
*il-8*	F	CGTTGAACAGACTGGGAGAGATG	64.9
	R	AGTGGGATGGCTTCATTATCTTGT	
*il-1β*	F	CGTGACTGACAGCAAAAAGAGG	59.4
	R	GATGCCCAGAGCCACAGTTC	
*tnf-α*	F	CTTCGTCTACAGCCAGGCATCG	63
	R	TTTGGCACACCGACCTCACC	
*zo-1*	F	ATCTCAGCAGGGATTCGACG	58
	R	CTTTTGCGGTGGCGTTGG	
*claudin-1*	F	CCAGGGAAGGGGAGCAATG	58.1
	R	GCTCTTTGAACCAGTGCGAC	
*claudin-4*	F	TAATCGCTATGGTGGGAGCC	57.7
	R	GCCCCGATCTCCATCTTCTG	

**Table 3:** Growth performance analysis of five groups of largemouth bass.

Indicator	SB0	ESB1	ESB2	ESB3	RSB
IBW (g)	29.76 ± 0.17	29.73 ± 0.04	29.75 ± 0.13	29.79 ± 0.27	29.66 ± 0.12
FBW (g)	82.96 ± 9.11 ^a^	84.74 ± 9.84 ^a^	90.29 ± 4.96 ^b^	81.57 ± 5.18 ^a^	83.78 ± 6.92 ^a^
WGR (%)	261.95 ± 1.39 ^a^	274.79 ±12.30 ^a^	303.53 ± 17.92 ^b^	273.73 ± 15.03 ^a^	274.03 ± 10.10 ^a^
SGR (%/d)	2.10 ± 0.01 ^a^	2.19 ± 0.09 ^a^	2.41 ± 0.13 ^b^	2.19 ± 0.12 ^a^	2.19 ± 0.08 ^a^
FC	1.20 ± 0.04 ^b^	1.10 ± 0.06 ^b^	0.93 ± 0.09 ^a^	1.09 ± 0.10 ^b^	1.11± 0.06 ^b^
SR (%)	100	100	100	100	100
FI (g)	2255.15 ± 8.13 ^a^	2249.87 ± 64.87 ^a^	2244.83 ± 22.96 ^a^	2236.07 ± 18.81 ^a^	2275.30 ± 24.42 ^a^
VSI (%)	7.17 ± 0.90 ^a^	6.98 ± 0.92 ^a^	7.14 ± 0.69 ^a^	7.32 ± 0.70 ^a^	7.32 ± 0.70 ^a^
HSI (%)	1.94 ± 0.55 ^a^	1.80 ± 0.33 ^a^	1.80 ± 0.34 ^a^	2.02 ± 0.42 ^a^	1.75 ± 0.43 ^a^

Notes: initial body weight (IBW), final body weight (FBW), weight gain rate (WGR), specific growth rate (SGR), feed coefficient (FC), feed intake (FI), viscerosomatic index (VSI), and hepatosomatic index (HSI). Different lowercase letters indicate significant differences between groups (*p* < 0.05), while the same lowercase letters indicate no significant differences (*p* > 0.05).

**Table 4:** Effects of dietary SB levels on the final whole-body composition of largemouth bass.

Indicators	SB0	ESB1	ESB2	ESB3	RSB
Moisture (%)	71.17 ± 0.19	71.52 ± 0.30	71.10 ± 1.05	71.40 ± 0.83	70.85 ± 0.25
Crude protein (%)	67.47 ± 0.86	64.54 ± 2.22	64.15 ± 1.72	62.90 ± 0.73	64.22 ± 0.74
Crude fat (%)	14.12 ± 0.03	14.41 ± 0.02	14.44 ± 0.03	12.32 ± 0.02	11.93 ± 0.02
Crude ash (%)	13.74 ± 0.69	13.85 ± 0.38	13.21 ± 0.84	13.08 ± 0.57	13.50 ± 0.29

**Table 5:** Effects of dietary SB levels on intestinal morphological parameters in largemouth bass.

Parameters	SB0	ESB1	ESB2	ESB3	RSB
Villus length					
Proximal intestine	664.45 ± 46.96 ^aC^	720.42 ± 44.33 ^bB^	754.33 ± 49.26 ^cC^	735.43 ± 43.99 ^bcC^	671.44 ± 34.73 ^aB^
Mid intestine	620.23 ± 47.07 ^bB^	601.63 ± 47.50 ^aA^	619.77 ± 47.98 ^bB^	697.08 ± 46.58 ^cB^	594.97 ± 29.93 ^aA^
Distal intestine	556.73 ± 41.13 ^aA^	597.84 ± 48.25 ^cA^	565.39 ± 43.14 ^aA^	592.38 ± 46.74 ^cA^	579.49 ± 41.56 ^bA^
Villus width					
Proximal intestine	105.99 ± 8.21 ^aA^	112.29 ± 8.90 ^bA^	111.35 ± 7.43 ^bA^	116.06 ± 8.96 ^cA^	110.50 ± 7.56 ^bA^
Mid intestine	119.43 ± 9.34 ^bB^	111.05 ± 6.36 ^aA^	115.37 ± 9.39 ^bA^	119.20 ± 9.33 ^bA^	118.40 ± 9.07 ^bB^
Distal intestine	129.84 ±9.22 ^dC^	121.59 ± 8.41 ^bB^	122.86 ± 9.18 ^bcB^	126.93 ± 9.67 ^bC^	113.66 ± 8.65 ^aA^
Muscularis thickness					
Proximal intestine	131.50 ± 19.85 ^bA^	115.87 ± 10.56 ^aA^	154.72 ± 12.18 ^dA^	118.92 ± 10.84 ^aA^	129.33 ± 13.02 ^bA^
Mid intestine	173.18 ± 13.42 ^bB^	154.49 ± 16.28 ^aB^	165.59 ± 11.71 ^bB^	171.88 ± 11.40 ^bB^	148.82 ± 14.71 ^aB^
Distal intestine	214.38 ± 14.87 ^cC^	157.06 ± 15.76 ^bB^	163.54 ± 15.95 ^bB^	168.81 ± 15.36 ^bB^	146.76 ± 12.79 ^aB^

Notes: Different lowercase letters indicate significant differences between different groups of the same intestinal area, while different uppercase letters indicate significant differences between the same group at the proximal, middle, and distal ends of the intestine.

**Table 6:** Effects of dietary SB on serum biochemical indicators in largemouth bass.

Indicators	SB0	ESB1	ESB2	ESB3	RSB
GLU (mmol/L)	5.39 ± 0.61	5.58 ± 0.31	5.99 ± 0.54	6.52 ± 1.59	6.27 ± 0.63
ALB (g/L)	14.33 ± 2.15	12.64 ± 1.36	14.05 ± 1.57	12.32 ± 1.36	13.49 ± 0.72
TP (g/L)	35.62 ± 4.20 ^b^	34.27 ± 4.67 ^ab^	35.43 ± 3.26 ^b^	29.89 ± 2.86 ^a^	33.44 ± 2.02 ^ab^
AST (U/L)	48.01 ± 14.36	52.70 ± 9.55	60.17 ± 14.51	57.91 ± 14.90	55.54 ± 15.78
ALT (U/L)	3.65 ± 1.51 ^a^	4.41 ± 0.97 ^ab^	4.85 ± 1.49 ^ab^	4.96 ± 0.73 ^ab^	5.49 ± 1.39 ^b^

Notice: alanine aminotransferase (ALT), aspartate aminotransferase (AST), albumin (ALB), glucose (GLU), and total protein (TP). Different lowercase letters indicate significant differences between groups (*p* < 0.05), while the same lowercase letters indicate no significant differences (*p* > 0.05).

**Table 7:** Effects of dietary SB levels on the alpha diversity of intestinal microbiota in largemouth bass.

Indicators	SB0	ESB1	ESB2	ESB3	RSB
Sobs	293.33 ± 28.04 ^b^	257.33 ± 99.05 ^ab^	208.00 ± 68.43 ^ab^	241.00 ± 128.51 ^ab^	167.50 ± 26.03 ^a^
Shannon	3.04 ± 0.16 ^b^	2.56 ± 0.45 ^ab^	2.06 ± 0.90 ^ab^	2.17 ± 0.90 ^ab^	1.94 ± 0.37 ^a^
Simpson	0.12 ± 0.04 ^a^	0.25 ± 0.09 ^ab^	0.32 ± 0.23 ^ab^	0.24 ± 0.16 ^ab^	0.29 ± 0.14 ^b^
Ace	299.69 ± 29.81 ^b^	260.60 ± 99.59 ^ab^	211.96 ± 68.90 ^ab^	245.91 ± 130.87 ^ab^	170.50 ± 25.15 ^a^
Chao	301.39 ± 31.45 ^b^	264.04 ± 98.50 ^ab^	212.58 ± 71.52 ^ab^	247.30 ± 127.45 ^ab^	172.58 ± 22.96 ^a^
Goods coverage	99.97	99.98	99.97	99.98	99.98

Notice: Different lowercase letters indicate significant differences between groups (*p* < 0.05), while the same lowercase letters indicate no significant differences (*p* > 0.05).

## Data Availability

The original contributions presented in this study are included in the article. Further inquiries can be directed to the corresponding authors.

## Abbreviations

ALB	albumin
ALT	alanine aminotransferase
AST	aspartate aminotransferase
CAT	catalase
DM	dry matter
DMPT	Dimethyl-β-propiothetin
ESB	encapsulated sodium butyrate
FBW	final body weight
FC	feed coefficient
FI	feed intake
GLU	glucose
HSI	hepatosomatic index
IBW	initial body weight
RSB	raw powder sodium butyrate
RT-qPCR	real-time quantitative polymerase chain reaction
SB	sodium butyrate
SGR	specific growth rate
SOD	superoxide dismutase
SR	survival rate
TP	total protein
VSI	viscerosomatic index
WGR	weight gain rate
